# The Long-Term Growth Impact of Refugee Migration in Europe: A Case Study

**DOI:** 10.1007/s10272-021-0951-3

**Published:** 2021-01-26

**Authors:** Gerrit Manthei

**Affiliations:** Institut für Finanzwissenschaft und Sozialpolitik, Albert-Ludwigs-Universität Freiburg, Bertoldstraße 17, 79098 Freiburg i. Breisgau, Germany

## Abstract

Many questions have been raised about the political and economic consequences of the recent surge in refugee immigration in Europe. Can refugee immigration promote long-term per capita growth? How are the drivers of per capita growth influenced by immigration? What are the policy implications of refugee immigration? Using an adjusted Cobb-Douglas production function, with labour divided into two complementary groups, this article attempts to provide some answers. By applying the model to current immigration data from Germany, this study finds that refugee immigration can lead to long-term per capita growth in the host country and that the growth is higher if refugee immigrants are relatively young and have sufficiently high qualifications. Further, capital inflows are a prerequisite for boosting per capita growth. These findings can inform policymakers of countries that continue to grapple with refugee immigration.

The issue of refugee immigration has dominated European politics for the past five years. The significant increase in immigration rates since 2015 and the mass migration into Southeast Europe, however, have not elicited uniform reactions across the continent. While most East European countries have been very restrictive, Germany and Sweden were initially more open to immigrants. This is because Germany, for example, can be characterised as a relatively tolerant society, and, initially, the majority of its population and the media were in favour of government policies (Haller, 2017). However, the subsequent change in public opinion (GfK Verein, 2018) led to policy revisions. Both Germany and Sweden have since adopted much stricter immigration regulations (*Migrationspaket* in Germany and temporary law of temporary residence status in Sweden). It seems unlikely that these countries will witness any large-scale immigration in the coming years.[1] However, given the alarming consequences of climate change (Perch-Nielsen et al., 2008) and the large wealth gap between Europe and North and Central Africa (Stark, 2017), one can reasonably assume that immigration rates in the future will be higher than previously estimated.[2]

Recognising the need for an in-depth analysis of immigration, many scholars have published studies on the social, political, demographic, economic and fiscal effects of refugee immigration in recent years. In Sweden, for example, most studies highlight the negative aspects of general and refugee immigration (Lundborg, 2013; Ruist, 2015), including the ones published before the 2015 surge. Similar findings have also been reported by studies that are not based on any individual country (Dustmann et al., 2017).

In Germany, some studies have focused on the positive economic effects of refugee immigration, especially those published in the first few months of the influx (Fratzscher and Junker, 2015). Later, however, papers on the negative economic effects of refugee immigration (van Suntum and Schultewolter, 2016), especially its effects on fiscal sustainability (Manthei and Raffelhüschen, 2018), became more pronounced. The present study attempts to offer a diverging viewpoint based on the theoretical assumption that population growth in absolute terms generally

1 Partly because of the social situation, partly because of the COVID-19 pandemic.

2 From a purely legal point of view, those who migrate for economic reasons are not refugees, but they share similarities with refugees in terms of age and qualification structures. Thus, the assumption that both groups have similar implications seems plausible.

**Gerrit Manthei**, University of Freiburg, Germany.

induces economic growth.[1] Accordingly, it examines the economic effects of refugee immigration by focusing specifically on per capita growth. It is important to add here that countries like Germany have a well-developed and comprehensive social system, in which the productive inhabitants support the less productive ones through tax-financed redistribution. Thus, negative per capita growth induced by refugees may place an additional burden on local taxpayers regardless of absolute economic growth.

The two main factors affecting the per capita growth effects of migration are age and qualification structure of the immigrants (Boubtane et al., 2016). *Ceteris paribus*, per capita growth can improve if the qualification structure of the refugees is better than that of the local population. Even a poor qualification structure among refugees can promote per capita growth provided a larger percentage of them are of working age compared to the native population, which then increases the labour force share of the total population (age structure effect). Another significant factor affecting per capita growth is capital mobility, particularly the increase of capital inflows from abroad, for example, via foreign direct investments (FDIs). If the increase in labour supply leads to a relative reduction in wages, economic theory suggests that the price of capital will rise and subsequently result in greater foreign investments (Samuelson, 1948) if factor price elasticity is sufficiently high. *Ceteris paribus*, this could lead to per capita growth. Apart from the above, other factors (e.g. state consumption and integration) can also affect per capita growth.

Interestingly, the growth effects of refugee immigration, whether per capita or absolute, have not been sufficiently researched. While the effects of general migration on growth have been extensively studied, those of refugee migration have not received much scientific attention. In light of future projections about refugee immigration, this topic is highly relevant not only from a scientific point of view but also from a political and social perspective.

Using an adjusted Cobb–Douglas production function with labour divided into two complementary groups, this article presents a two-step quantitative analysis of the long-term per capita growth effects of refugee migration. The research aims to determine whether the effects are mainly positive or negative, to assess the impact of individual drivers of growth and to derive policy implications. This article focuses on Germany because the country has accepted the highest number of refugees in Western Europe since 2015, and it represents a midpoint within Europe in terms of geography, per capita growth and the welfare state system.

1 The expected rise in demand alone would lead to growth. Further, each additional employee increases the country's economic output.

## Theoretical model

According to the Cobb–Douglas production function, the output (GDP in this study) is dependent on the production factors: labour and capital. Labour usually refers to the number of workers in an economy or their working hours. Capital is typically defined as all the assets in a national economy (i.e. cash and financial assets as well as buildings, land and machinery). Further, government consumption is considered in this study to better account for integration costs.

Taking the above factors into account, GDP ($Y_t$) in every year *t* is given by:

$$Y_t = \beta \cdot c_{S,t} \cdot K_{S,t}^{\alpha_1} \cdot K_{P,t}^{\alpha_2} \cdot L_{WC,t}^{\alpha_3} \cdot L_{BC,t}^{\alpha_4}. \quad (1)$$

Here $\beta$ is the total factor productivity, which serves as a scaling factor to scale the model's output to the actual GDP. $c_{S,t}$ denotes the impact of state consumption on GDP and includes, for example, integration costs. Capital is divided into two categories. The first category, state capital stock ($K_{S,t}$), is mostly subject to the constraints of investment and depreciation (Equation 4) and is only indirectly influenced by immigration. The second category, private capital stock ($K_{P,t}$), inter alia, depends on the size of the labour force in the national economy (Equation 7) and is therefore directly exposed to the effects of migration.

To capture the growth effects of refugee migration in a meaningful way, the labour factor needs to be differentiated according to productivity. Since productivity is more difficult to quantify in data lacking a migration context, the analysis uses qualification levels as they are strongly linked to productivity (Becker, 1962). Accordingly, the labour force is divided into two groups: an above-average productive group (white-collar workers), with excellent qualifications, and a less productive group (blue-collar workers), with lower qualifications. To consider the possible migration-related wage effects, wages are used instead of the number of workers. Thus, $L_{WC,t}$ is the sum of all the wages of white-collar workers, and $L_{BC,t}$ is that of blue-collar workers. Depending on the qualification structure of the immigrants, the ratio of blue- to white-collar workers can change and, following the theory of supply and demand, affect relative labour prices (wages).

The coefficients $\alpha_1$, $\alpha_2$, $\alpha_3$ and $\alpha_4$ are fixed over time and define the impact of each type of capital and wage factor on the output. The sum of all four coefficients is 1. $\alpha_1$ and

$\alpha_2$ represent the share of GDP that is derived from gross profit. They show the influence of the two capital stocks (state and private) on nominal GDP. $\alpha_3$ and $\alpha_4$ denote the share of GDP derived from the labour force. These coefficients together capture the impact of the sum of all wages on GDP.

The following equation accounts for state consumption:

$$c_{S,t} = \left(\frac{C_{S,t}}{C_{S,t\text{-}1}}\right) \cdot \frac{C_{S,0}}{Y_0}, \tag{2}$$

where $c_{S,t}$ is the scalar of state consumption, and ($C_{S,0}$ / $Y_0$) scales the impact of this scalar to GDP. The absolute consumption of the state is defined as

$$C_{S,t} = \bar{C}_S + P_t \cdot \bar{c}_S^{flex} + (1 - \sigma) \cdot E_{BI,t}, \tag{3}$$

with $\bar{C}_S$ as a fixed level of state consumption. It does not vary with the size of the population $P_t$, because some expenditures, such as defence, are relatively inelastic to changes in population size. Most other expenditures are calculated with a constant per capita sum $\bar{c}_S^{flex}$. The rest of the state consumption is driven by integration costs $E_{BI,t}$. This includes direct integration costs for services such as food, shelter, medical aid and language courses provided to immigrants. It also accounts for spending on unemployment, under-age immigrants, social assistance for the elderly and the costs incurred on deportation/voluntary departures. This paper treats integration costs as state consumption and assumes that the state finances these integration costs by cutting down its consumption or its investments.[2] However, the inclusion of integration costs under state consumption does not negatively affect the latter, as the category of expenditures is irrelevant to GDP. On the other hand, cuts in investments to pay for integration costs [$(1 - \sigma) \cdot E_{BI,t}$] do increase consumption. The factor $\sigma$, which takes a value between 0 and 1, denotes how much of the integration costs are covered by cuts in state consumption.

The state capital stock is estimated as follows:

$$K_{S,t} = (K_{S,t\text{-}1} - K_{S,t\text{-}1} \cdot \bar{q}_A + I_{S,t\text{-}1}) \cdot lk_t. \tag{4}$$

Each year, the capital stock depends on that of the previous year ($K_{S,t\text{-}1}$) and on the development of the relative price of labour to capital ($lk_t$; Equation 6). Further, it decreases by the fixed depreciation rate $\bar{q}_A$ and increases with the state's investment ($I_{S,t\text{-}1}$), which is calculated by

$$I_{S,t} = K_{S,t} \cdot \bar{q}_I - (1 - \sigma) \cdot E_{BI,t}. \tag{5}$$

It is assumed that each year, a fixed quota ($\bar{q}_I$) is invested by the state. $\bar{q}_I$ and $\bar{q}_A$ are ideally fixed with the same value, so that the state capital stock decreases over time if investment cuts are used to finance integration costs $(1 - \sigma)$. In the short term, $Y_t$ increases for all $\sigma < 1$ as short-term consumption offsets long-term investment in the state capital stock because of $\alpha_1 < 1$. Subsequently, a negative relationship develops between immigration and the state capital stock because immigrants benefit from public capital spending without having contributed to it through, for example, tax or social contribution payments (Piras, 2011). With refugees unable to bring in their capital,[3] their immigration, or more precisely their integration and the associated costs, will lead to a long-term decrease in state capital and present a hindrance to growth.

The development of the relative price of labour to capital is given by:

$$lk_t = \frac{\left(\frac{LF_{t\text{-}1}}{K_{S,t\text{-}1} + K_{P,t\text{-}1}}\right)^{\alpha_3+\alpha_4}}{\left(\frac{LF_0}{K_{S,0} + K_{P,0}}\right)^{\alpha_3+\alpha_4}}. \tag{6}$$

$lk_t$ accounts for relative price changes of capital to labour to meet the principle of supply and demand. For example, an increase in the size of the labour force ($LF_t$), *ceteris paribus*, leads to a decrease in wages and an increase in the price of capital.

Analogously, the development of the relative price of capital to labour ($kl_t$) is given by:

$$kl_t = \frac{\left(\frac{K_{S,t\text{-}1} + K_{P,t\text{-}1}}{LF_{t\text{-}1}}\right)^{\alpha_1+\alpha_2}}{\left(\frac{K_{S,0} + K_{P,0}}{LF_0}\right)^{\alpha_1+\alpha_2}}. \tag{7}$$

Private capital is strongly affected by the size of the labour force and by the development of the relative price of labour to capital:

$$K_{P,t} = (\overline{K_{FP}} + LF_t \cdot \overline{k_{LF}}) \cdot lk_t. \tag{8}$$

While $\overline{K_{FP}}$ is a fixed share of the private capital stock that

2 Borrowing, another possible alternative to finance these costs, is excluded from the model. For host countries that usually follow a strict policy of balanced budgets like Germany, this modelling seems realistic.

3 On average, refugees pay €7,100 per person to flee to Germany (Federal Office for Migration and Refugees, 2016), which may possibly constitute their entire mobile capital.

is independent of labour force changes, $\overline{k_{LF}}$ is a fixed amount of per capita capital that each member of the labour force holds or attracts. Private capital is computed in this way because domestic firms may borrow money to satisfy higher demand for goods. But with a higher supply of labour, and the consequent increase in the factor price for capital, borrowing money in the host country will become more expensive than borrowing from abroad. This could stimulate capital inflows. In addition, the host country is favourably placed to attract long-term FDIs from the rest of the world. As the economic theory of factor price equalisation (Samuelson, 1948) states, an open economy with a relatively high factor price tends to encourage an inflow of the respective factor.

The sum of all white-collar workers' wages is calculated by

$$L_{WC,t} = LF_{WC,t} \cdot w_{WC,t}. \tag{9}$$

$w_{WC,t}$ is the average yearly wage of a white-collar worker, and $LF_{WC,t}$ is the total number of white-collar workers. This yearly wage depends on the yearly wage in the base year ($w_{WC,0}$), the development of the ratio of blue- to white-collar workers and the relative price of labour in the host country:

$$w_{WC,t} = w_{WC,0} \frac{\left(\frac{BC_t}{WC_t}\right)^{\alpha_4}}{\left(\frac{BC_0}{WC_0}\right)^{\alpha_4}} \cdot kl_t. \tag{10}$$

The first quotient captures the development of the ratio of blue- to white-collar workers. In each year, the ratio of blue- to white-collar workers is calculated in relation to their ratio in the base year.[4] Such modelling implies that any change in the ratio has a direct impact on the wages of the workers. For example, if the proportion of blue-collar workers among immigrants is higher than that in the host country, immigration can lead to a relative increase in the wages of white-collar workers. If the ratio of total capital stock to total workforce increases, relative to the base year, the price of labour increases and thus the wages.

The number of blue- and white-collar workers in each period, as well as of $P_t$, depends on three factors: demographics, migration and integration. The present analysis employs a population projection model to account for demographic changes and a future decrease in Germany's total labour force, owing to the double ageing process.[5] However, the latter does not interfere with the analysis of migration-induced effects, because it is factored into all the calculations.

The second factor – migration – is modelled by dividing the number of immigrants in every year based on age and wage (two wage groups). Emigration is modelled by estimating the number of emigrants across population groups and by taking into account the significantly higher emigration of the non-integrators, because statistics clearly show that foreigners constitute a larger share of emigrants (Federal Statistical Office of Germany, 2019a).

Integration is the third factor that affects the number of blue- and white-collar workers. New refugees of working age (or who will attain working age within the projection period) who will not emigrate during the projection period will typically integrate first. This trend is modelled by assuming a logarithmic assimilation process (integration) with an individual duration for each wage group, while accounting for unemployment.

## Data and scenarios

### Descriptive statistics and data

This case study considers 2014 as the base year, as Germany witnessed a significant increase in refugee immigration in the following year. The main sources of data are the national accounts of Germany (Federal Statistical Office of Germany, 2016a) and the survey of income and expenditure, EVS (Research Data Centre of the Statistical Offices of the Federal States, 2015).

In 2015 and 2016, the average age of immigrants entering Germany was 31 years (Federal Statistical Office of Germany, 2019b), while that of the German population in 2014 was approximately 44 years (Federal Statistical Office of Germany, 2016b). Further, the proportion of immigrants aged 65 or below was 98.5% (Federal Statistical Office of Germany, 2019b), while the proportion of the German population under 65 was only 78% (Federal Statistical Office of Germany, 2016b). Thus, *ceteris paribus*, immigration could have initiated per capita growth by increasing the working age population.

This paper considers workers with an income equal to or higher than 150% of the national average as white-collar workers. The analysis uses income for the 2014 labour force instead of qualification levels as it is directly linked

4 The equations of the wage bill of all blue-collar workers and of their yearly wage is designed analogously to Equations 9 and 10.

5 Growing life expectancy rates and lower birth rates.

Figure 1
**Net immigration trend in Germany and future projections**

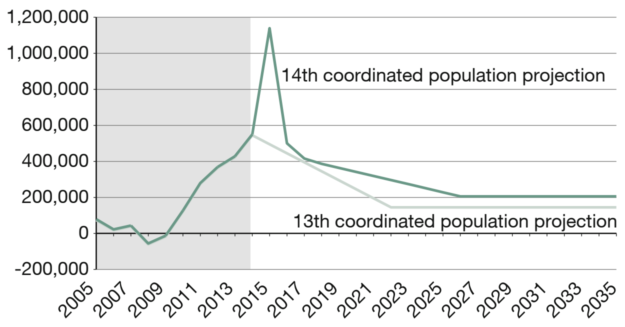

Note: The grey area on the left side marks the pre-projection period and serves to illustrate the changing immigration in recent years.

Source: Author's own illustration based on Federal Statistical Office of Germany (2015, 2019b, 2019c).

Table 1
**Overview of the main scenarios**

| | Pessimistic | Base | Optimistic |
|---|---|---|---|
| Integration time (white-collar) | 12 years | 9 years | 6 years |
| Integration time (blue-collar) | 9 years | 6 years | 3 years |
| Share of white-collar qualifications | 17% | 21.6% | 24.3% |
| Share of blue-collar qualifications | 83% | 78.4% | 75.7% |

Source: Author's own elaboration.

to the necessary wage sums of Equation 1. According to EVS, the initial distribution of workers in Germany in 2014 was as follows: 24.3% white-collar and 75.7% blue-collar. Of the foreigners living in Germany before the 2015 immigration, 21.6% were white-collar, and 78.4% were blue-collar workers. Equations 8 to 11 suggest that a high share of blue-collar workers among foreigners (and refugees) can, if future refugee immigrants have the same income or qualification distributions as the foreigners already living in Germany, lead to a decrease in the wages of blue-collar workers and an increase in that of white-collar workers.

To measure the net growth effects of refugee immigration, two migration trends are developed (Figure 1). First, a hypothetical migration movement without high immigration numbers, plotted with the help of data obtained from the 13th coordinated population projection (Federal Statistical Office of Germany, 2015). The second migration trend is derived from the actual migration figures between 2015 and 2018 (Federal Statistical Office of Germany, 2019b) and is then linearly adjusted to long-run net immigration of 206,000 as in the second immigration scenario of the 14th coordinated population projection (Federal Statistical Office of Germany, 2019c). A *ceteris paribus* comparison of the two migration trends allows for an estimation of the net effects of refugee immigration, because of the 1.1 million net immigrants in 2015 (Federal Statistical Office of Germany, 2016c), about 890,000 were refugees (Federal Ministry of the Interior, 2016).

## Main scenarios

Three scenarios are hypothesised as part of the first step of the quantitative analysis.[6] Subsequently, per capita net-growth effects are estimated with the help of a base scenario, which includes the basic assumption about immigrants' workgroup distribution (21.6% vs 78.4%) derived from the dataset and probable integration times (Table 1).

An average integration time of six years is considered for blue-collar workers, following the work by Manthei and Raffelhüschen (2018). The integration process for white-collar workers is set at nine years, which is 1.5 times longer than for that of blue-collar workers. This is due to the fact that it is extremely important to speak the host country's language in jobs requiring high qualification levels. Further, high-skilled immigrants may first work in jobs below their qualification level to gain financial security. Moreover, the process of acknowledging the qualifications achieved in the home country by German standards, which is required by many jobs, may be time-consuming.

Because the assumptions of integration time and qualification distribution are riddled with uncertainty, two other scenarios are presented – one highly pessimistic and one highly optimistic (Table 1). These scenarios serve as the lower (pessimistic scenario) and upper limit (optimistic scenario) of a result corridor.

In the optimistic scenario, the qualification distribution of immigrants is assumed to be identical to that of the natives in the host country. The share of white-collar workers in the pessimistic scenario is based on the UNESCO International Standard Classification of Education (ISCED11-A) of refugee immigrants in Germany.[7] According to data

6 The second step is a sensitivity analysis to assess the impact of single variables.

7 Education is segmented into 10 levels in the 2011 version (ISCED11). This paper uses the categorisation attainment (A) for individuals who graduated in their respective segment (ISCED11-A).

Figure 2
**Yearly growth effects (per capita) in the base scenario**

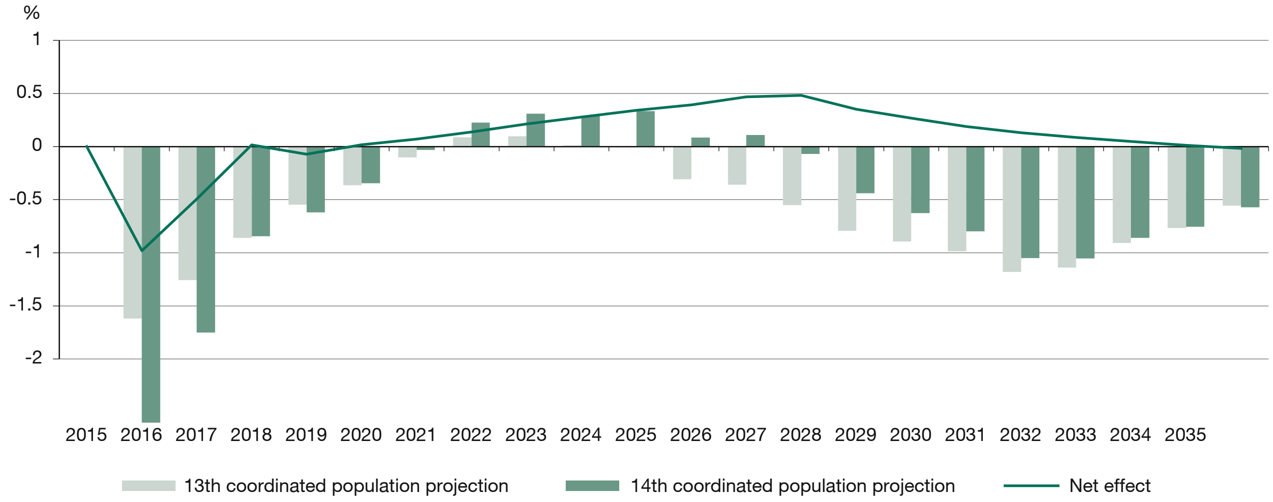

Note: The zigzag course in 2017/2018 is data-driven as the number of emigrants dropped sharply in 2017 (in the 14th coordinated population projection).

Source: Author's estimations.

from the German Institute of Economic Research (2017), about 17% of the refugees entering Germany in 2016 were highly qualified (ISCED11-A level 6 or higher).

## Results

### Main scenario results

Figure 2 shows the yearly per capita growth effects of both migration trends in the base scenario. As expected, in the first few years, when an assumed integration process delays the newly migrated refugees from entering the labour market directly, per capita growth effects are negative. They are also negative under both migration trends for most years of the projection period and only become slightly positive between 2021 and 2026. While this is mainly due to (e)migration in the early years, the negative growth effects after 2026 are primarily the result of demographic changes following the retirement of the baby boomer generation. As the 14th coordinated population projection includes higher emigration rates, the negative per capita growth effects in the second migration trend (dark green bars) are stronger at first. This is why the net effect of refugee immigration (green line) is also negative in the initial projection years. The break-even point is reached in the year 2021, after which the per capita net growth effects of refugee immigration remain positive until the year 2026. Subsequently, the net effect declines until the per capita growth effects of both migration trends converge. These results suggest that refugee immigration in Germany could indeed have a positive effect on its per capita growth in some years.

Figure 3 displays the aggregated per capita growth effect across the years of the projection period. The net effect (dashed line) reaches a break-even point in 2026 and stabilises with a long-term positive growth effect of approximately 1.70%. This confirms the results presented in Figure 2, suggesting that refugee immigration could lead to long-term per capita growth even with a below-average qualification structure. However, it is important to note that the assumptions described in the 'main scenarios' above are subject to uncertainty. Therefore, the net per capita growth effects of the pessimistic and optimistic scenarios, in relation to the base scenario, are of interest, too. As expected, the curve of the pessimistic scenario (grey line) is below that of the base scenario. While a longer integration period shifts the break-even point to the right, it is only delayed by around two years and not by three years, as could be inferred by this scenario's assumptions. The long-term net growth of 1.33% is lower than that of the base scenario, which highlights the importance of the qualification structure of the refugee immigrants.

The curve of the optimistic scenario (green line) lies above that of the base scenario. Here, the break-even point is reached about three years earlier than in the base case (in 2023). Additionally, long-term growth is the highest at

Figure 3

**Main scenarios: Aggregated net growth effects (per capita)**

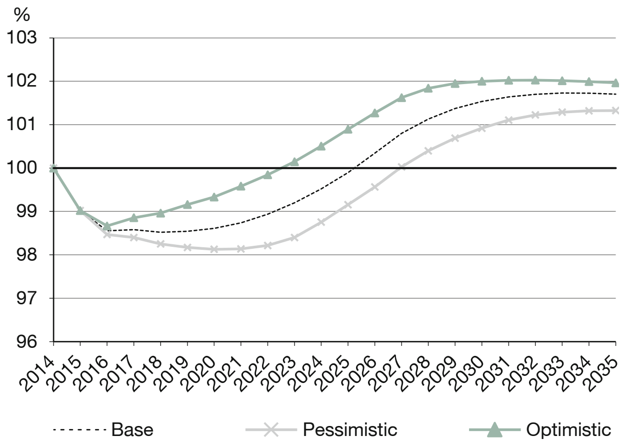

Source: Author's estimations.

1.96% at the end of the projection period. Thus, the results of the optimistic scenario confirm the implications above.

### Sensitivity analysis

The second step of the quantitative analysis assesses the impact of individual variables. To examine the effect of each variable, the above three scenarios are remodelled fixing the concerned variable, for example, when analysing the influence of state capital and foreign capital inflows on per capita growth. Alternatively, the same data is used for refugees and residents, for example, for the respective age or qualification structure. Figures 4.A-H show the results in comparison with those from the first step of the quantitative analysis.

The immigrants' age structure has a strong influence on the per capita growth trend (Figure 4.A). Without such a favourable age structure of refugees, per capita growth will be significantly lower in all three scenarios, by about one percentage point each (thus, half as strong). Weaker but significant effects exist for the qualification structure (Figure 4.B), the wage effects (Figure 4.G), and the relative price development (Figure 4.H). The integration time has no effect on the absolute growth number, but on its growth path (Figure 4.C). State consumption and the state capital stock have negligible effects (Figures 4.D-E).

Without migration-induced capital inflows from abroad (Figure 4.F), long-term per capita growth turns negative.[8] This finding underscores the importance of capital inflows, without which a negative correlation can be expected between per capita growth and refugee immigration, even if the qualification structure of refugees is the same as that of the natives (optimistic scenario: -0.62%).

## Conclusion

Refugee immigration is currently one of the most crucial topics in European political discourse, and it is likely to remain so in the foreseeable future. The economic consequences associated with refugee immigration can significantly affect the lives of the European population. This study examines the long-term per capita growth effects of refugee immigration with the help of an augmented Cobb–Douglas production model and a two-step quantitative analysis that explored a range of economic scenarios.

The results indicate that refugee immigration can lead to long-term per capita growth. Key to this development is the age structure of refugees and, to a slightly lesser degree, their qualification structure. The length of time needed by refugees to integrate mainly determines the time required to reach the break-even point. Interestingly, the results show that private capital stock has the greatest impact on per capita growth. Without a migration-related increase in the available private capital stock in the host country, positive per capita growth is unlikely, even under optimistic assumptions. In fact, the per capita economic output could drop significantly.

As the proposed model does not contain assumptions that are specific to Germany, the results of the case study may be generalised to other countries affected by refugee immigration. But the effects of refugee immigration on the capital stock in the host country have not yet been conclusively researched. Thus, it is difficult to definitively assert that refugee immigration leads to long-term per capita economic growth in the host country.

Nonetheless, three political implications arise from these results. First, promoting the quick and successful integration of refugees will increase per capita growth. Second, granting permanent residence permits to young and highly qualified individuals will ensure their positive contributions in the long run. And third, reducing barriers to capital inflows is in everyone's best interest as it is a prerequisite for per capita growth.

8 Because of the large population, the overall economic growth, without capital inflow, remains positive in the base scenario (0.83%).

Figure 4

**Sensitivity analysis: Aggregated net growth effects (per capita)**

Source: Author's estimations.
